# A Behavioral Economics–Electronic Health Record Module to Promote Appropriate Diabetes Management in Older Adults: Protocol for a Pragmatic Cluster Randomized Controlled Trial

**DOI:** 10.2196/28723

**Published:** 2021-10-27

**Authors:** Hayley M Belli, Andrea B Troxel, Saul B Blecker, Judd Anderman, Christina Wong, Tiffany R Martinez, Devin M Mann

**Affiliations:** 1 Division of Biostatistics, Department of Population Health Grossman School of Medicine New York University New York, NY United States; 2 Division of Healthcare Delivery Science, Department of Population Health Grossman School of Medicine New York University New York, NY United States; 3 Department of Medicine Grossman School of Medicine New York University New York, NY United States; 4 Medical Center Information Technology New York University Langone Health New York, NY United States

**Keywords:** diabetes, behavioral economics, electronic health records, clinical decision support, randomized controlled trial, pragmatic

## Abstract

**Background:**

The integration of behavioral economics (BE) principles and electronic health records (EHRs) using clinical decision support (CDS) tools is a novel approach to improving health outcomes. Meanwhile, the American Geriatrics Society has created the Choosing Wisely (CW) initiative to promote less aggressive glycemic targets and reduction in pharmacologic therapy in older adults with type 2 diabetes mellitus. To date, few studies have shown the effectiveness of combined BE and EHR approaches for managing chronic conditions, and none have addressed guideline-driven deprescribing specifically in type 2 diabetes. We previously conducted a pilot study aimed at promoting appropriate CW guideline adherence using BE nudges and EHRs embedded within CDS tools at 5 clinics within the New York University Langone Health (NYULH) system. The BE-EHR module intervention was tested for usability, adoption, and early effectiveness. Preliminary results suggested a modest improvement of 5.1% in CW compliance.

**Objective:**

This paper presents the protocol for a study that will investigate the effectiveness of a BE-EHR module intervention that leverages BE nudges with EHR technology and CDS tools to reduce overtreatment of type 2 diabetes in adults aged 76 years and older, per the CW guideline.

**Methods:**

A pragmatic, investigator-blind, cluster randomized controlled trial was designed to evaluate the BE-EHR module. A total of 66 NYULH clinics will be randomized 1:1 to receive for 18 months either (1) a 6-component BE-EHR module intervention + standard care within the NYULH EHR, or (2) standard care only. The intervention will be administered to clinicians during any patient encounter (eg, in person, telemedicine, medication refill, etc). The primary outcome will be patient-level CW compliance. Secondary outcomes will measure the frequency of intervention component firings within the NYULH EHR, and provider utilization and interaction with the BE-EHR module components.

**Results:**

Study recruitment commenced on December 7, 2020, with the activation of all 6 BE-EHR components in the NYULH EHR.

**Conclusions:**

This study will test the effectiveness of a previously developed, iteratively refined, user-tested, and pilot-tested BE-EHR module aimed at providing appropriate diabetes care to elderly adults, compared to usual care via a cluster randomized controlled trial. This innovative research will be the first pragmatic randomized controlled trial to use BE principles embedded within the EHR and delivered using CDS tools to specifically promote CW guideline adherence in type 2 diabetes. The study will also collect valuable information on clinician workflow and interaction with the BE-EHR module, guiding future research in optimizing the timely delivery of BE nudges within CDS tools. This work will address the effectiveness of BE-inspired interventions in diabetes and chronic disease management.

**Trial Registration:**

ClinicalTrials.gov NCT04181307; https://clinicaltrials.gov/ct2/show/NCT04181307

**International Registered Report Identifier (IRRID):**

DERR1-10.2196/28723

## Introduction

### Background

Behavioral economics (BE) is a field that combines the disciplines of psychology and economics to provide insight into how humans often fail to behave as perfectly rational agents [1-10]. The challenges for individuals of carefully weighing the costs and benefits in decision-making and arriving at optimal choices can be described via a variety of BE principles. A nudge is a BE-based tool that seeks to provide positive reinforcement and influence the behavior and decision-making of individuals or groups. Nudges are implemented in a variety of contexts, and their recent application in medicine to improve health outcomes has grown in popularity [11-17].

The specific system used to deliver nudges, as well as the environment in which they are deployed, will ultimately influence their success in improving health outcomes. One potential mode for nudge delivery is via electronic health record (EHR) technology. Clinicians interact daily with EHRs, which guide nearly all aspects of clinical care, including documentation, ordering, data review, and communication. Furthermore, providers may interact with clinical decision support (CDS) tools when accessing patient EHRs; these tools provide alerts and suggestions and redirect clinical behavior. EHRs and CDS tools therefore serve as an ideal platform for delivering nudges designed to influence clinician behavior, leading to improved patient care [18-22].

While the delivery of nudges through EHR and CDS tools shows great promise, the specific disease subtypes and environments in which nudges are most effective at influencing clinician behavior and subsequent patient health outcomes have yet to be determined. Nudges embedded within the EHR have been shown to improve processes of care, including reducing the rate of inappropriate antibiotic prescriptions for acute respiratory infections [13], increasing influenza vaccination rates [23], encouraging completion of high-value cancer screening tests [24], and increasing guideline-concordant statin prescribing [25]. There is, however, limited evidence supporting the effectiveness of nudges to positively influence chronic disease management, especially among older adults with type 2 diabetes mellitus.

Choosing Wisely (CW) is an American Board of Internal Medicine initiative to identify unnecessary tests, treatments, and procedures [26]. The American Geriatrics Society released 10 guidelines in 2013 (revised in 2015), the third of which promotes less aggressive glycemic targets and reduction in pharmacologic therapy for older adults with type 2 diabetes [27-29]. Providers may be unaware of these guidelines, resulting in excessive glycemic indices in older adults [30].

Herein, we describe the design of a pragmatic, cluster randomized controlled trial to test the effectiveness of a toolbox of nudges embedded within the EHR and utilizing CDS tools to promote appropriate diabetes management in older adults (ClinicalTrials.gov identifier: NCT04181307). Specifically, the behavioral economics–electronic health record (BE-EHR) intervention aims to reduce the overtreatment of older adults with diabetes per the CW guideline. The proposed study seeks to test the effectiveness of the newly developed, user-tested, refined, and previously pilot-tested BE-EHR intervention at promoting appropriate diabetes management in older adults.

### Prior Work

We conducted a pilot study of the BE-EHR module intervention (ClinicalTrials.gov identifier: NCT03409523) in 5 clinics across the New York University Langone Health (NYULH) system [31]. The BE-EHR module consists of 6 nudge components, 2 of which were launched in 2 NYULH sites from June 12, 2018, until October 23, 2018, 4 (including the first 2 nudges) were launched in 5 American Geriatrics Society sites on October 24, 2018, and the 2 nudges each deployed on December 10, 2018, and April 8, 2019, respectively. All nudges were active in the NYULH EHR system, Epic (Epic Systems Corporation), through October 22, 2019. Despite the 6 BE-EHR components being introduced at varying time points across an approximately 10-month interval, we observed a 5.1% increase in CW compliance rates between the 16-week interval just prior to the launch of the first 2 nudges and the final 16 weeks of the pilot study [31].

Interpretation of the pilot study results, however, was limited. First, the intervention was launched in only 5 practices that were not selected at random. Second, due to the deployment of the nudges over an approximately 10-month period, all 6 BE-EHR components were active simultaneously for approximately 6 months, making the long-term effects of the entire intervention toolbox difficult to observe. Finally, only ~71% of the patient population had a return visit or new HbA_1c_ (hemoglobin A_1c_) lab test at least 90 days after an initial test, suggesting that changes in CW compliance were not measurable in almost one-third of the study participants.

Thus, while the pilot study was successful in the development, user testing, and implementation of the BE-EHR module within the NYULH EHR system, a full-scale, sufficiently powered randomized controlled trial of longer duration is necessary to estimate the effectiveness of the intervention at reducing the overtreatment of older adults with type 2 diabetes.

## Methods

### Setting

The cluster randomized controlled trial will be conducted in NYULH primary care and endocrinology clinics, which span the greater New York City area and include 2 sites in Florida. The NYULH system provides an ideal setting for this pragmatic study design due to its diverse patient sociodemographics and large population of older patients. Patient inclusion and administration of the intervention will occur using the NYULH EHR system, Epic. The study was approved by the Institutional Review Board at NYULH.

### Eligibility Criteria

Study inclusion criteria require patients to be aged ≥76 years with type 2 diabetes as defined on the patient’s “problem list” or “encounter diagnosis” within the EHR. Exclusion criteria include not taking medication to treat diabetes, an allergy to the medication metformin, and an estimated glomerular filtration rate of <30.

### Randomization

The study will randomize 66 eligible clinics to 1 of 2 groups: (1) BE-EHR module + standard care or (2) standard care only. Clinics eligible for randomization must be active Epic users and have had at least 1 patient encounter in the year prior for which the above patient eligibility criteria were met.

We conducted stratified randomization to ensure balance with respect to practice size and location. Practice size was measured by counting the number of eligible patients with an encounter at each NYULH site in 2019. The median number of eligible patients seen at each of the randomization-eligible practices in 2019 was 57 patients per site. Randomization was therefore stratified by clinics with fewer than or equal to 57 eligible patients in 2019 or greater than 57 eligible patients in 2019. Sites were also stratified by location into 1 of 2 groups: (1) practices located within Manhattan or Brooklyn or (2) practices outside of these 2 New York City boroughs. Randomization assignments according to this plan were generated by an unblinded study statistician using the software R v3.6.3 (The R Foundation for Statistical Computing) [32].

### Study Design and Enrollment

**Figure 1:**
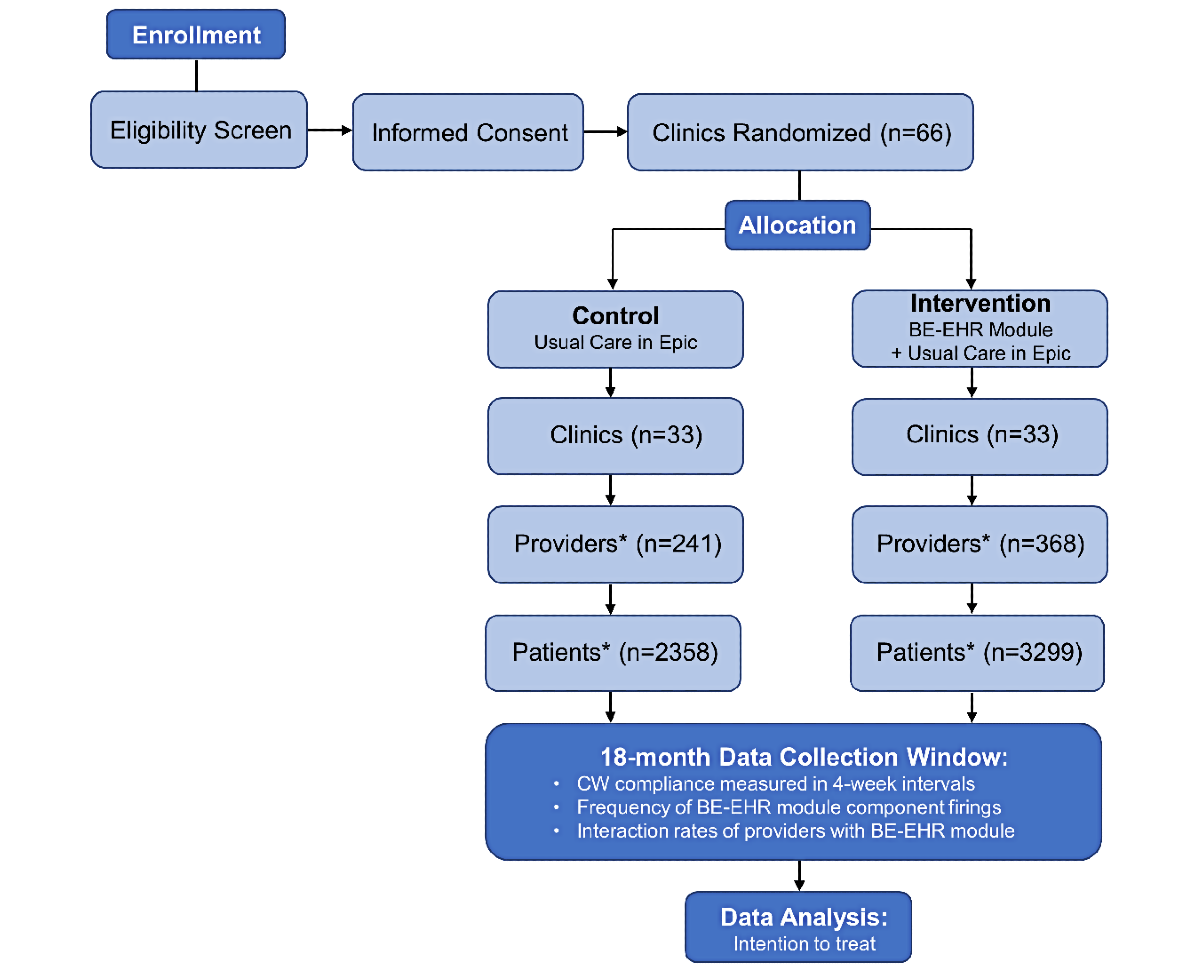
Flow chart of the study design. A total of 66 clinics across New York University Langone Health were randomized 1:1 to the behavioral economics–electronic health record (BE-EHR) module + standard care (intervention) or standard care only (control) after meeting eligibility criteria and informed consent requirements. *The number of providers and patients per arm is an initial estimate as of October 12, 2020, based on eligible patient-provider encounters from the prior 18 months. Due to the dynamic nature of the study being embedded within the EHR, we expect providers and patients to enter and leave the study over the 18-month duration window. CW: Choosing Wisely.

The study design is a pragmatic, investigator-blind, cluster randomized controlled trial. A total of 66 NYULH clinics were randomized 1:1 to receive either a 6-component BE-EHR module + standard care intervention or standard care only (control) for a duration of 18 months. The study design is unique in that clinicians rather than patients within each practice will receive the intervention through their interaction with the NYULH Epic system during any eligible patient encounter (eg, in person, telemedicine, lab order, prescription refill, etc). Individual patients were not recruited; rather, we obtained a waiver of consent from the NYULH Institutional Review Board, and patients of physicians at participating clinics who met eligibility criteria were included. Therefore, patients at all eligible primary care, family care, and endocrinology clinics within the NYULH system were automatically recruited and enrolled in the study (Figure 1).

Prior to randomization, eligible practices were identified, and site directors were informed of the study and offered the opportunity to ask questions and to opt out of participation. The study design is investigator-blind in that the principal investigators will be blinded to the assignment of clinics to either the intervention or control arm, and all interim analyses will be presented to the blinded study team labeled only as “arm 1” and “arm 2.” Only the study statistician and the Medical Center Information Technology Epic data management team will be unblinded, as will the clinicians receiving intervention-based alerts while interacting with Epic during an eligible patient encounter. The Data and Safety Monitoring Board will also receive information specific to the intervention and control arms.

### Intervention

#### Overview

The BE-EHR module intervention contains 6 components or “nudges,” each leveraging principles grounded in BE theory. Each component was developed to interface with NYULH’s EHR system. Providers at the 33 clinics randomized to the intervention arm will receive elements of the BE-EHR module either when activated during an eligible patient encounter or according to a monthly dissemination schedule.

To appropriately tailor the intervention to the patient population and reflect the CW guideline, we developed an algorithm to categorize patients into 1 of 3 life expectancy categories: low, medium, and high. The algorithm incorporated a patient’s current age and gender, and used a weighted scoring approach for the number of chronic conditions [33,34], along with previously developed life expectancy tables from Medicare beneficiary data [35]. The full description of this algorithm can be found in the supplementary information provided by Belli et al [31]. This life expectancy algorithm was programmed into the NYULH EHR system to drive content firing based on a patient’s life expectancy categorization.

#### Design and User Testing

We employed a pragmatic, user-centered approach to develop the 6 components of the BE-EHR module for implementation into the NYULH EHR [36,37]. Full details of the design and user testing of the intervention during the pilot study phase can be found in Belli et al [31]; briefly, this entailed semistructured interviews with key informants; two 2-hour design thinking workshops; and site visits to 2 of the 5 clinics, including in-person observation of clinician use and interaction with the module in a live clinical setting.

#### BE-EHR Module Components

A detailed description of each BE-EHR module component, including the BE principles utilized and which key aspects of the user testing and feedback aided in the design, is provided below. Corresponding visualizations of each nudge in Epic can be found in the supplementary materials of Belli et al [31].

##### Nudge 1: Tailored Advisory

The “tailored advisory” BE-EHR module component activates noninterruptively in Epic during a clinician-patient encounter for any CW-noncompliant patient. It consists of an alert window that describes appropriate treatment guidelines for older adults given the individual’s life expectancy categorization. Although a response is not required, clinicians may interact with the alert by clicking the “Agree with recommendation. Action taken” button, or by selecting the “Clinically inappropriate. Please explain” option, with space for free-text comments. Clinicians may also choose to suppress future activations of the tailored advisory nudge for a particular patient for half a year (182.5 days) with either of these acknowledgments. The optional nature of the alert, as well as the ability to suppress future activations, resulted directly from the user-design process. The tailed advisory nudge utilizes BE principles, including framing [5,38], social norming [39], suggesting alternatives [5], affirmation [40,41], emotional appeal [42], and accountable justification [43-45].

##### Nudge 2: Refill Protocol

The “refill protocol” is an alert window that appears in the refills section of Epic whenever a refill for diabetes medication is generated for study-eligible older adults. The alert suggests that providers order metformin as an alternative for patients who are not already taking the medication, or to consider refilling at a lower dose or not at all for patients who are already taking metformin. The provider may leave comments, but this is not required. During the user-testing phase, clinicians preferred the ability to leave comments as an optional action. The refill protocol nudge utilizes BE principles, including framing [5,38], social norming [39], suggesting alternatives [5], affirmation [40,41], emotional appeal [42], and accountable justification [43-45].

##### Nudge 3: Preference List

The “preference list” is implemented at the system level by providing an automatic default list, with metformin displayed at the top of the list of medications for “First-line Type 2 Diabetes.” Orders for nonmetformin medications are not restricted, which was in line with clinician preferences during user testing. This nudge uses the BE defaults principle [14,46-48].

##### Nudge 4: Lab Result

The “lab result” nudge is an alert window that appears in the lab results section describing appropriate treatment guidelines for older adults whenever there is a new HbA_1c_ lab result for a CW-noncompliant patient. Features of the design, including the red text for those patients out of range and tabular formatting, resulted from the user-testing feedback. The alert remains active for 7 days following the result in Epic. BE principles utilized include framing [5,38], social norming [39], suggesting alternatives [5], and emotional appeal [42].

##### Nudge 5: Peer Comparison

The “peer comparison” nudge is the first of 2 nudges sent outside of the Epic system. Once per month, the peer comparison nudge is sent via a secured Microsoft Outlook account with the email subject line: “Message from the desk of Dr. [Insert Practice Director Name].” The email content includes 3 graphics displaying (1) CW compliance for the individual provider, (2) CW compliance for the clinician’s practice site, and (3) CW compliance across all NYULH practices. The provider then receives either a “negative” or “positive” version of the accompanying text, depending on whether the clinician’s CW compliance rate is above or below the rate of their respective practice. The graphic design of this nudge and positive versus negative text versions were iteratively refined after feedback from both clinicians and health services researchers. The peer comparison nudge utilizes BE principles, including social norming [39] and peer comparisons [49].

##### Nudge 6: Campaign

The “campaign” is the second nudge to alert clinicians outside of the Epic system; it serves the goal of bringing awareness to the CW guideline. The campaign was developed through a series of design workshops that included clinicians, researchers, and health services experts; game show–themed prototypes emerged and were ultimately user tested. The final campaign toolkit included 3 game show–themed animations inspired by *The Price is Right*, *Jeopardy*, and *Who Wants to Be a Millionaire*, as well as a flashcard deck that quizzes physicians on CW best practices. There are multiple versions among each of the 4 campaign themes that vary according to the 3 life expectancy categories and information provided. Clinicians receive at random a version of a campaign theme every month. The campaign utilizes BE principles, including gamification and competition [49].

### Data Collection and Study Outcomes

The primary study outcome will be patient-level CW compliance. To measure CW compliance, patients will first be categorized into 1 of 3 life expectancy groups:

1. High life expectancy: healthy older adults with a limited number of comorbidities and a life expectancy of >10 years; HbA_1c_ target range of 7%-7.5%
2. Medium life expectancy: older adults with a moderate number of comorbidities and a life expectancy of 3+ to 10 years; HbA_1c_ target range of 7.5%-8%
3. Low life expectancy: older adults with multiple comorbidities and a life expectancy of ≤3 years; HbA_1c_ target range of 8%-9%.

Per the CW guideline, patients within each of these life expectancy categorizations will then be categorized as either CW compliant or noncompliant depending on their measured HbA_1c_ relative to the target range within their respective life expectancy category.

The following set of equations measure CW compliance and noncompliance for each life expectancy category:

1. High life expectancy:
  - CW compliance = ratio of eligible patients with HbA_1c_ range: 
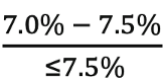
  - CW noncompliance = ratio of eligible patients with HbA_1c_ range: 
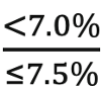
2. Medium life expectancy:
  - CW compliance = ratio of eligible patients with HbA_1c_ range: 
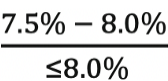
  - CW noncompliance = ratio of eligible patients with HbA_1c_ range: 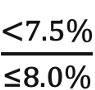
3. Low life expectancy:
  - CW compliance = ratio of eligible patients with HbA_1c_ range: 
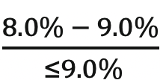
  - CW noncompliance = ratio of eligible patients with HbA_1c_ range: 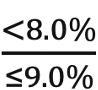

Secondary study outcomes will include the frequency of activation rates of the individual BE-EHR module components longitudinally; provider interaction with each intervention component, measured by frequency of clicks and workflow sequences within the NYULH EHR system; and email read receipts from the peer comparison and campaign nudges.

### Statistical Analysis

#### Power

We used data collected during the pilot phase to inform the power calculation for the full-scale randomized controlled trial. In that study, a moderate increase of 5.1% in CW compliance was detected [31]. We used data from the pilot phase to adequately power the randomized controlled trial to detect a similar reduction of approximately 5 percentage points in the rate of CW noncompliance. These data from the pilot phase suggested an intraclass correlation coefficient (a measure of the degree of additional correlation among providers within the same practice) of 0.01. With an average of 10 providers per practice site, this yields a design effect of 1.09. Using the available pilot data, and assuming a type I error rate of 0.05 for a 2-sided test, 66 eligible NYULH practices (33 per arm) will provide 93% power to detect an effect size of 0.1. This power calculation supports the evaluation of the effectiveness of the 6-component BE-EHR module intervention as a combined toolkit; to discern the effectiveness of individual nudges, a prohibitively large trial would be required. In addition, providers will often receive multiple nudges at various time points and in differing order, making the evaluation of the effectiveness of individual BE-EHR module components challenging.

#### Analytic Plan

We will begin all analyses with descriptive summary statistics and graphical displays of all variables. All analyses will be performed using the software R v3.6.3 [32].

The primary study outcome will be patient-level CW compliance, which will be modeled using a 3-level logistic mixed-effects model. A binary indicator of patient-level CW compliance will serve as the dependent variable. Treatment group, time, and the interaction of the 2 variables will serve as the primary fixed effects, where time will be measured using indicator variables for each 4-week interval. The model will also include a patient-level random effect, provider-level random effect, and practice-level random effect to account for patients seen by providers nested within clinics. Although randomization should obviate the need for adjustment, we will explore whether it is necessary to adjust for covariates, including gender, age, life expectancy category, and total number of patient visits, the latter of which may capture a potential dose-response effect of exposure to the intervention.

Secondary outcomes, including activation of each module component and provider interaction with the BE-EHR module within Epic, will be reported as frequency counts overall and stratified by gender, age, life expectancy, and use of metformin versus nonmetformin medications. We will also analyze any free-text comments provided within the accountable justification components of the tailored advisory and refill protocol nudges to look for patterns and any association with CW compliance.

We will furthermore look at CW compliance rates among patients whose providers only received a single nudge or particular combination of nudges. Although statistical power will likely be limited for these comparisons, this exploratory analysis will provide clues as to which individual BE-EHR components may be more or less effective at improving patient CW compliance and guide future research in this area.

Finally, safety information will be collected, including the frequency of in-patient hospitalizations, emergency department visits, and deaths across the control and intervention arms.

## Results

We randomized a total of 66 practices, with 33 clinics in each arm. The breakdown of practices by strata is shown in Table 1.

All 6 BE-EHR module intervention components were activated in the NYULH EHR on December 7, 2020. The study will run for a duration of 18 months with a possibility for extension if patient clinic visit activity is affected by the COVID-19 pandemic. All final results will be disseminated via publications, conference proceedings, and presentations.

**Table 1:** Clinics randomized by location and number of eligible patients.

Location and sample size	Control (n=33), n (%)	Intervention (n=33), n (%)
Manhattan/Brooklyn, ≤57 patients	10 (30)	7 (21)
Manhattan/Brooklyn, >57 patients	7 (21)	6 (18)
Outside Manhattan/Brooklyn, ≤57 patients	6 (18)	9 (27)
Outside Manhattan/Brooklyn, >57 patients	10 (30)	11 (33)

## Discussion

This study is a novel, pragmatic randomized controlled trial that incorporates BE nudges into the EHR and CDS tools to promote CW guideline adherence in a chronic condition, namely type 2 diabetes. The BE-EHR module was designed after an extensive literature review of studies that have utilized BE nudges to improve clinical outcomes. Design of the BE-EHR module also incorporated behavioral change theory models, as well as a variety of BE principles. The extensive user testing process that the interdisciplinary study team undertook led to the development of an intervention that aims to reduce cognitive load on physicians through seamless integration into the NYULH EHR system, while allowing physicians the opportunity to interact with various BE-EHR module components only if they choose. Examples include leaving comments, following links to additional material regarding the CW guideline, or receiving a list of CW-noncompliant patients with whom they have interacted during a clinical encounter in the past month.

Furthermore, this study is innovative in that it tests the use of BE nudges in promoting clinical deprescribing. The research is also unique in that the intervention targets clinicians rather than the patients themselves. Furthermore, studying a population of elderly patients who have been living with type 2 diabetes and other chronic conditions for potentially long durations poses an interesting behavioral change problem that the study team seeks to address using BE nudges within the EHR.

Limitations of the study include testing of the BE-EHR module as a whole rather than evaluating individual components. However, a toolbox of BE nudges has yet to be proven effective in the context of treating type 2 diabetes, making this study an important first step in understanding the effectiveness of BE nudges for promoting appropriate type 2 diabetes management and the potential of BE nudges to lead to positive clinical outcome among other chronic conditions. If effective, further work will evaluate which elements of the toolbox are most impactful.

Furthermore, the study team attempted to reduce alert fatigue via a user-centered design that incorporated feedback from clinicians to help minimize the burden of the alerts. Additionally, some of the alerts were passive or noninterruptive, thus reducing their contribution to alert fatigue [50]. However, we acknowledge that any clinician interaction with CDS tools and EHRs poses a threat of clinician burnout [51,52]. Hence, we measure the primary outcome of CW compliance longitudinally in 4-week increments to ultimately estimate the duration of the intervention’s effectiveness.

A third limitation is that the CW guideline may evolve over time, especially with the recently demonstrated benefits of sodium/glucose cotransporter-2 inhibitors (SGLT2i) and glucagon-like peptide 1 (GLP-1) for diabetes and cardiovascular diseases [53]. Fortunately, all 6 components of the BE-EHR module are highly adaptable to changing guidelines, as well as to other CDS tools outside of the NYULH EHR system.

Results will estimate the effectiveness of the BE-EHR module in improving CW compliance, as well as provide insight into clinician interaction with the BE-EHR module. This study will therefore not only provide answers to fundamental questions regarding the effectiveness of BE nudges at promoting appropriate diabetes management, but will also guide future research aimed at optimizing the timing and location of BE nudges within the EHR and CDS tools, and inform other chronic conditions for which administering BE nudges using digital health tools may lead to improved clinical outcomes.

Based on pilot study results [31], we hypothesize that the BE-EHR module will increase patient-level CW compliance. In addition to testing the effectiveness of the BE-EHR module at promoting appropriate diabetes management in older adults, this study will also yield results on provider interaction with the module. This valuable information will provide insights into the real-time clinical workflow while BE nudges are being activated, providing useful information about the visibility of BE nudges to providers.

## Appendix

Multimedia Appendix 1 CONSORT-EHEALTH checklist (V1.6.1).

## Abbreviations

BE: behavioral economics
BE-EHR: behavioral economics–electronic health record
CDS: clinical decision support
CW: Choosing Wisely
EHR: electronic health record
NYULH: New York University Langone Health
SGLT2i: sodium/glucose cotransporter-2 inhibitors
GLP-1: glucagon-like peptide 1
